# Clothing increases the risk of indirect ballistic fractures

**DOI:** 10.1186/1749-799X-8-42

**Published:** 2013-11-25

**Authors:** David C Kieser, Debra J Carr, Sandra CJ Leclair, Ian Horsfall, Jean-Claude Theis, Michael V Swain, Jules A Kieser

**Affiliations:** 1 Medical Corps, New Zealand Defence Force, Wellington 5045, New Zealand; 2 Orthopaedic Registrar Surgical Sciences, Orthopaedic Surgery, Health Sciences, Dunedin School of Medicine, University of Otago, 364 Leith Walk, PO Box 6458, Dunedin 9016, New Zealand; 3 Impact and Armour Group, Department of Engineering and Applied Science, Cranfield Defence and Security, Defence Academy of the United Kingdom, Shrivenham, Wiltshire SN6 8LA, UK; 4 Department of Health Sciences, University of Angers, Angers 49100, France; 5 Sir John Walsh Research Institute, University of Otago, 364 Leith Walk, Dunedin 9016, New Zealand

**Keywords:** Fracture, Ballistic, Non-contact, Indirect, Temporary cavity, Clothing

## Abstract

**Background:**

Current literature has shown the mechanism of how indirect fractures occur but has not determined what factors increase the risks of such fractures. The objective of this study is thus to determine the effect of clothing and soft tissue thickness on the risk of indirect fracture formation.

**Methods:**

Twenty-five fresh red deer femora embedded in ballistic gelatine were shot with varying distances off their medial cortex with a 5.56 × 45 mm North Atlantic Treaty Organization (NATO) bullet while being filmed with a slow-motion video. We compared the effect of two different gelatine depths and the effect of denim cloth laid onto the impact surface of the moulds.

**Results:**

Bullet passage in thinner moulds failed to cause fracture because the bullet exited the mould before a large expanding temporary cavity was produced. Clothing dramatically altered the size and depth of the expanding cavity, as well as increased lateral pressures, resulting in more severe fractures with greater bullet distances from the bone that can cause fracture.

**Conclusions:**

Clothing increases the risk of indirect fracture and results in larger, more superficial temporary cavities, with greater lateral pressures than are seen in unclothed specimens, resulting in more comminuted fractures. Greater tissue depth affords the 5.56 × 45 mm NATO a chance to yaw and thus develop an enlarging temporary cavity that is sufficient to cause fracture.

## Background

Gunshot injuries continue to be a major cause of death and morbidity worldwide with over 500,000 people killed each year and more than 1.5 million injured [1]. This injury principally affects a younger working population in both the military and civilian populations [2,3]. More recently, interest in gunshot wounds has increased, not only because of the wars in Iraq and Afghanistan but also because of increasing civilian gunshot wounds [3,4]. There are over 115,000 projectile injuries annually in the USA, 80,000 of these are caused by gunshot injuries and 45% of these present with a fracture [4,5].

When a bullet impacts a target, a shock wave is generated that advances through the tissues but does not significantly affect the bone because its duration is too short [6-8]. The bullet itself crushes and lacerates tissues within its path but, in addition, causes lateral pressures that force tissues apart, thus creating a temporary cavity [9]. While it is principally the bullet that fractures the bone, a significant current discussion has focussed on the effect of the expanding cavity in fracture formation [10]. In recent papers, it has been shown that the expansion of the temporary cavity causes fractures in near-miss gunshot trauma, where the bullet traverses the soft tissues but never contacts the bone directly [10].

Callender and French first described these unique injuries in 1935 [11]. However, since then, only limited research has been presented on the topic [10,12,13]. However, recent publications by Dougherty et al. [12] and Kieser et al. [10] have shown that these fractures of the femur are directly related to the size and proximity of the expanding temporary cavity to the bone [10,12]. Kieser et al. [10] found that despite a .44 in. (magnum semi-jacketed hollow-point Remington MG43) bullet’s slower velocity, the fact that it expands on impact results in a larger, more superficial temporary cavity than 5.56 × 45 mm (North Atlantic Treaty Organization (NATO) SS109), 762 × 39 mm (FMJ mild steel core, M43, Russian, Factory 71, 1984) or 9 × 19 mm (FMJ, DM1 1A1B2) projectiles [10]. This resulted in the fracture of deer femora, embedded in 20% (by mass) ballistic gelatine, even when the bullet passed no closer than 30 mm from the bone. In comparison, the 5.56 × 45 mm was seen to produce fracture only up to 10 mm off the bone, while the 762 × 39 mm and 9 × 19 mm failed to cause fracture.

Damage to tissues is often claimed to directly relate to the transfer of kinetic energy [14]; however, this is only one facet determining energy severity, and it may be more prudent to assess how the bullet disrupts the tissue [15] or, more importantly, where within the tissues this energy is deposited [16,17]. It has long been known that bullets that are designed to fragment or expand create significant injuries. The British Indian Army employed this technique in the 1890 s to saw off the tips of jacketed .303 bullets, exposing the softer lead core. This was the infamous dumdum bullet that resulted in devastating injuries [18] because it increased bullet expansion and fragmentation on impact, resulting in a more superficial temporary cavity, ensuring the bullet imparted its kinetic energy to the victim, rather than ‘just passing though’ [19,20]. However, due to the significant morbidity associated with fragmenting and expanding bullets, The Hague Convention of 1899 banned their use in armed conflict [21]. Since then, multiple amendments and refinements have been made to the agreement as different bullet designs have been introduced to produce more severe injuries. However, no research has studied the effect of body habitus, morphology or clothing on wounds sustained by ‘non-expanding’ bullets. This study assessed the factors of tissue depth and clothing in the development of indirect fracture for a bullet currently used in Afghanistan by NATO forces: the 5.56 × 45 mm (NATO SS109).

## Methods

The research performed during this experiment was conducted under the ethical approval of the University of Otago Animal Ethics Committee (No. 68/11) and performed according to the principles of ethical research practice, as described in the eighth edition of the *Guide for the Care and Use of Laboratory Animals* published by the National Academy of Sciences, The National Academies Press, Washington, D.C.

Twenty-three adult female red deer (*Cervus elaphus*) rear femora were obtained from a local processing plant on the day of slaughter (average length 276 mm (270–281 mm), mid-diaphyseal width 27 mm (25–29 mm)). Their legs had been disarticulated through the hip and the soft tissues stripped, leaving the periosteum intact, within an hour of slaughter. These samples were immediately refrigerated at 4°C and kept moist with saline-soaked gauze. All samples were tested within 4 days after being embedded in 20%, 250B ballistic gelatine (Weishardt International, Graulhet, France), made by mixing lukewarm water to 8 kg of gelatine in a cement mixer. Ballistic gelatine (20%) was utilised because of its similarity to the human muscle and it is often quoted as the standard NATO concentration [22,23]. No specific calibration testing was performed on the individual blocks because previous studies have shown that the variability within and between batches of gelatine made in our laboratory is minimal [24]. Twenty-one samples were embedded to a depth of 80 mm in rectangular containers of 180 (depth) × 180 (breadth) × 300 (length) mm, and two were embedded, again at 80 mm, in the same containers, except with a depth of 120 mm. All femora were positioned so that the anterior cortex faced the surface of the gelatine and their long axis paralleled that of the gelatine.

The samples were left to solidify overnight at room temperature (8°C). Six of the thicker rectangular mould samples were draped on their anterior surface with a single layer of denim fabric (typically used to manufacture jeans; 3 × 1 twill, 410 g/m^2^) that had been laundered six times according to Section 8 of *BS EN ISO 6330/A1: 2009—Textiles—Domestic Washing and Drying Procedures for Textile Testing* and dried flat according to Section 10C of the same standard [25]. A further two samples were draped in a similar fashion but with two layers of denim (Table 1).

**Table 1:** Separation of samples into groups

	**Unclothed**		**Clothed**	
**Group**	**Thick mould**	**Thin mould**	**Single layer**	**Double layer**
Number	13	2	6	2

Note that all clothed samples were shot through a thick mould.

All blocks were positioned 10 m from, and with their anterior cortex facing, a number 3 Enfield pressure housing, fitted with an appropriate barrel to fire a 5.56 × 45 mm (NATO SS109) bullet. Bullets were shot with varying distances medial to the medial cortex of the bone.

A pressure sensor (Kistler Type 7005 sn113590, Winterthur, Switzerland) with a 0–600-bar range was inserted into a 1-cm^3^ excised area of gelatine on its medial side at the same depth as the bone and height as the bullet trajectory. This was connected to a charge amplifier (Kistler Type 5041) in an Imatek C3008 data acquisition system (Knebworth, UK) and detected the peak pressure before being dislodged from the gelatine.

A slow-motion camera (Phantom V12, Vision Research, Wayne, NJ, USA; 40,000 frames per second) was positioned on the lateral side of the block and a 45° mirror positioned above the sample, giving synchronised images in the sagittal and axial planes.

Bullet impact velocity and exit velocity were recorded using a Doppler radar and confirmed with three sky-screen chronographs (MS Instruments, Orpington, UK). Energy transfer to the block was calculated from the change in pre- and post-impact kinetic energy of the bullet, assuming no loss or gain of mass of the bullet, using the formula

$${\mathrm{Ek}}_{\text{change}}=1/2m{v_{i}}^{2}–1/2m{v_{f}}^{2}$$

where Ek is the kinetic energy (J), *m* is the mass of the bullet (kg), *v*_i_ is the initial velocity (m/s) and *v*_f_ is the final velocity (m/s).

After testing, each block was dissected to assess the permanent cavity and the effect on the bone and periosteum. The video was analysed for temporary cavity dimensions, potential contamination, bone displacement and deformation. Pressure recordings were analysed in accordance with their distance from the bullet tract.

## Results

Fracture was produced in four unclothed thick samples (one was a direct impact on the bone and was therefore excluded), four single-layer clothed samples, one double-layer clothed sample and none in the thin samples.

**Figure 1:**
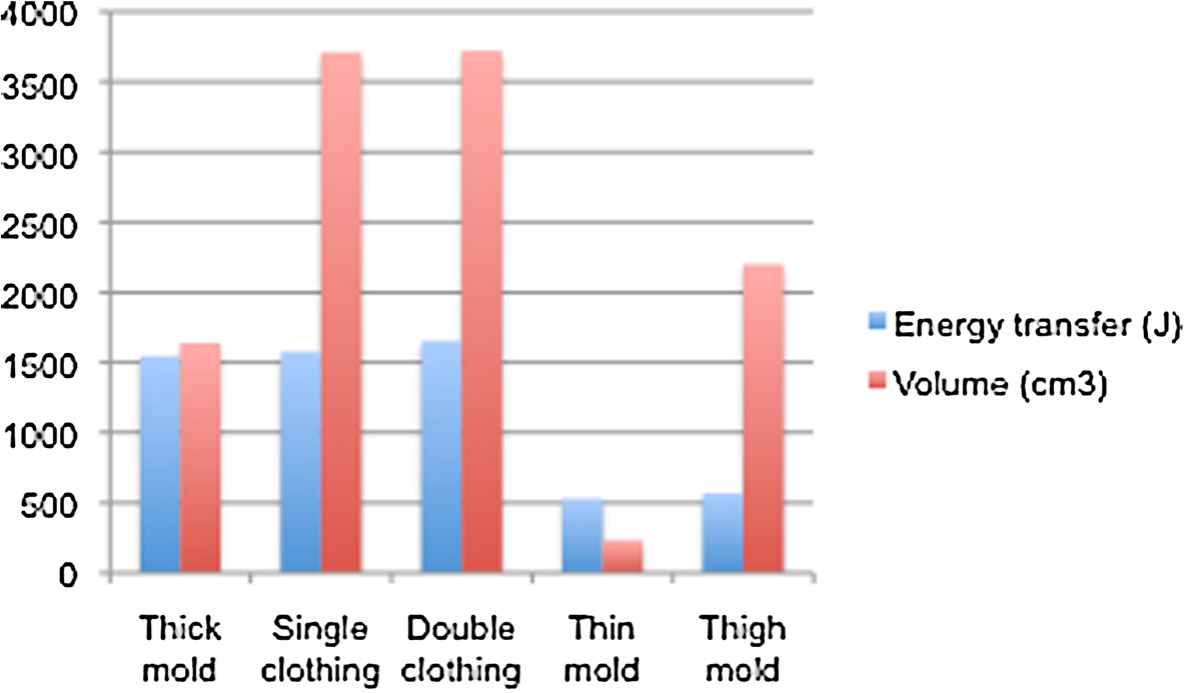
The average energy transferred to each mould and corresponding temporary cavity volume.

The average pre-impact velocity of the bullet was 970 m/s (range 959–980 m/s), with an average energy transfer to the sample of 1,560 J (1,307–1,874 J) for the thick unclothed mould, 1,578 J (1,453–1,784 J) for the single layer of clothing, 1,654 J (1,640–1,668 J) for the double layer and 533 J (527–539 J) for the thin rectangular mould (Figure 1).

The morphology of the temporary cavity changed with the different samples. For the unclothed rectangular moulds, the bullet passed to an average depth of 100 mm before starting to yaw. For the thin mould, the bullet passed through the mould before yawing sufficiently to develop a significant temporary cavity. In these samples, the temporary cavity expanded to an average maximum diameter of 60 mm (55–65 mm) with an average volume of 235 cm^3^ (150–340 cm^3^). For the thicker rectangular mould, the bullet’s maximum yaw was seen at an average depth of 140 mm (80–170 mm), consistent with Bowen and Bellamy [6]. This resulted in a spherical temporary cavity with an average maximal diameter of 140 mm (110–200 mm) and an average volume of 1,640 cm^3^ (900–4390 cm^3^).

By contrast, the bullets passing through clothing rapidly yawed and occasionally fragmented (three samples), developing a far more superficial and cylindrical temporary cavity commencing at an average depth of 20 mm (0–60 mm). For the single layer of clothing, the temporary cavity enlarged to an average maximal diameter of 150 mm (130–200 mm) and an average volume of 3,710 cm^3^ (2,790–6,600 cm^3^). For the double clothing, the temporary cavity expanded to an average maximal diameter of 160 mm (150–170 mm) and an average volume of 3,720 cm^3^ (3,220–4,220 cm^3^) (Figure 1).

**Figure 2:**
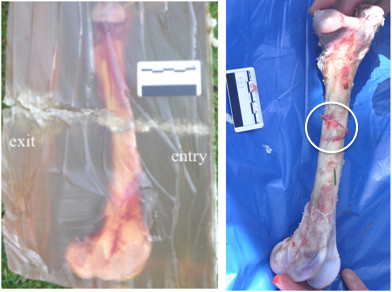
**Indirect fracture in an unclothed thick rectangular specimen.** The fracture was caused by a 5.56 × 45 mm NATO bullet passing 10 mm medially to the medial cortex of a femur. Note the simple undisplaced wedge-shaped fracture pattern (*circle*).

**Figure 3:**
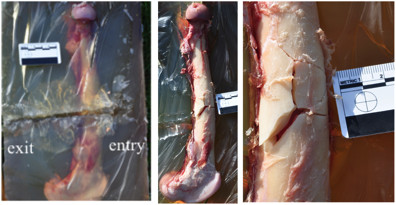
**Indirect fractures in a thick rectangular specimen, clothed in a single layer of denim material.** The fracture was caused by a 5.56 × 45 mm NATO bullet passing 10 mm medially to the medial cortex of a femur. Note the characteristic wedge-shaped fracture but with significantly more comminution and the more superficial permanent cavity.

The injuries were associated with indirect wedge-shaped fractures occurring with bullet passage of up to 10 mm off the bone for the thick rectangular undressed moulds. No fracture was produced in the thin moulds. Clothing accounted for a fracture occurring with bullet passage of up to 20 mm off the mould, but no observable difference was seen with the number of layers of clothing. When comparing the clothed and unclothed samples shot 10 mm off the bone, the fractures were more comminuted for the clothed samples, with an average of four fragments in comparison to one, respectively (Figures 2 and 3).

**Figure 4:**
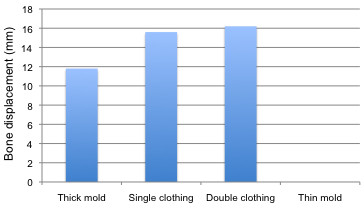
Maximal bone displacement with bullet passage 10 mm from the bone.

In addition, maximal bone displacement for the thick undressed moulds at 10 mm was on average 11.8 mm (range 10.2–13.5 mm), for the thin mould was 0 mm, for the single layer of clothing was 15.6 mm (range 15.3–15.9 mm) and for the double layer of clothing was 16.2 mm (Figure 4).

The permanent cavity morphology mirrored that of the temporary cavity. For the undressed thick rectangular moulds, a long narrow canal of an average of 80 mm from the entry site expanded into an enlarging cavity with radial tears of up to 70 mm. For the thin mould, this cavity started to enlarge at around 80 mm but only developed radial tears averaging 30 mm before exiting. For the clothed samples, the permanent cavity’s enlargement was seen to occur far more superficially at an average depth of 10 mm (range 0–30 mm) for the single layer of clothing and 20 mm (range 0–40 mm) for the double layer. The size of the radial tears was on average 90 mm for both samples (range 70–100 and 80–100 mm, respectively).

**Figure 5:**
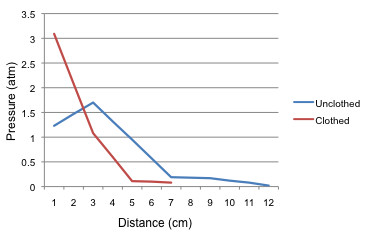
**Pressure exerted on the bone by the bullet passage at varying distances from the bone.** The clothed and unclothed samples are compared.

The pressure recording revealed the highest pressures for closer bullet tracts, which decayed with increasing distance from the transducer, consistent with Kieser et al. [10]. Complete and reliable pressure recordings were only available to compare clothed (single layer) and unclothed thick rectangular samples. This revealed significantly higher local pressures (Figure 5) and rates of pressure transduction in the clothed samples.

## Discussion

In this study, we used embedded deer femora in ballistic gelatine to show that increased soft tissue results in greater wounding potential of the 5.56 × 45 mm NATO bullet. Here we found that the 5.56 × 45 mm NATO failed to yaw significantly, before exiting the thinner mould. A significant temporary cavity thus failed to develop and the femur remained uninjured. This may explain why the humerus, with less soft tissue than the thigh, seems to be spared of indirect fractures [11].

The study also showed that clothing resulted in more rapid bullet yaw and occasionally fragmentation. This produced greater lateral pressures, with larger and more superficial temporary and permanent cavities. This correlated with both a greater risk of indirect fracture and a greater severity of those fractures produced. Clothing may also increase infection rates in gunshot injuries by being drawn into the wound tract and acting as a nidus for infection [26]. If this is combined with our findings of more severe injuries, the risk of infection is likely to be markedly increased if a victim is shot through clothing [27,28]. The reason why multiple layers of clothing were not significantly different to a single layer is unknown but may be related to the small sample size in this study.

Limited to non-deforming bullets, weapon manufacturers have continued to increase bullet velocity to produce more significant wounds and hence greater stopping power. This is based on the idea that greater initial kinetic energy affords greater potential energy transfer [29]. An additional and more sinister incentive may lie in the fact that greater velocity bullets have a greater chance of fragmenting on impact [21,30]. In this study, we used a 5.56 × 45 mm NATO, as this is the most common allied calibre currently being used in warfare, and found that 37.5% of these ‘non-fragmenting’ bullets fragmented when passing through clothing. Despite low numbers limiting this study, further research into the effect of clothing on bullet performance is warranted.

This study is limited by a number of factors: principally, its low numbers and restricted comparison of multiple layers of clothing to a single layer. Additionally, its sample design uses ballistic gelatine because of its similar density to that of the human skeletal muscle [22] and deer femur because of its similar morphology to that of the human femur. However, this design fails to account for fascial planes, skin and variations within the soft tissue densities, elasticity and cohesiveness present within the living human thigh. Finally, the permanent cavity size was described by the size of the radial tears, rather than accurately assessing the volume. This method lacks consistency as tear size may vary at random. Further research should aim to obviate these shortcomings.

## Conclusions

Clothing increases the risk of indirect fracture and results in larger, more superficial temporary cavities, with greater lateral pressures than are seen in unclothed specimens, resulting in more comminuted fractures. Greater tissue depth affords the 5.56 × 45 mm NATO a chance to yaw and thus develop an enlarging temporary cavity that is sufficient to cause fracture and may explain why the humerus is relatively spared from this injury.

## Competing interests

The authors declare that they have no competing interests.

## Authors’ contributions

DCK was the principal investigator. DJC and IH were integral in the study design, implementation and analysis. SCJL carried out the data analysis and writing, while JCT, MVS and JAK performed the data interpretation and critical revision. All authors read and approved the final manuscript.
